# Dendritic cell modification as a route to inhibiting corneal graft rejection by the indirect pathway of allorecognition

**DOI:** 10.1002/eji.201242914

**Published:** 2012-12-04

**Authors:** Adnan Khan, Hongmei Fu, Lee Aun Tan, Jennifer E Harper, Sven C Beutelspacher, Daniel F P Larkin, Giovanna Lombardi, Myra O McClure, Andrew J T George

**Affiliations:** 1 Section of Molecular Immunology, Department of Medicine, Imperial College London, Hammersmith HospitalLondon, United Kingdom; 2 Moorfields Eye HospitalLondon, United Kingdom; 3 Medical Research Council Centre for Transplantation, King's College London, Guy's HospitalLondon, United Kingdom; 4 Jefferiss Research Trust Laboratories, Wright-Fleming Institute, Department of Medicine, Imperial College London, St. Mary's HospitalLondon, United Kingdom

**Keywords:** Corneal transplantation, Dendritic cells, Gene therapy, Tolerance induction, Transplantation tolerance

## Abstract

Dendritic cell (DC) modification is a potential strategy to induce clinical transplantation tolerance. We compared two DC modification strategies to inhibit allogeneic T-cell proliferation. In the first strategy, murine DCs were transduced with a lentiviral vector expressing CTLA4-KDEL, a fusion protein that prevents surface CD80/86 expression by retaining the co-stimulatory molecules within the ER. In the second approach, DCs were transduced to express the tryptophan-catabolising enzyme IDO. CTLA4-KDEL-expressing DCs induced anergy in alloreactive T cells and generated both CD4^+^CD25^+^ and CD4^+^CD25^−^ Treg cells (with direct and indirect donor allospecificity and capacity for linked suppression) both in vitro and in vivo. In contrast, T-cell unresponsiveness induced by IDO^+^ DCs lacked donor specificity. In the absence of any immunosuppressive treatment, i.v. administration of CTLA4-KDEL-expressing DCs resulted in long-term survival of corneal allografts only when the DCs were capable of indirect presentation of alloantigen. This study demonstrates the therapeutic potential of CTLA4-KDEL-expressing DCs in tolerance induction.

## Introduction

Dendritic cells (DCs) not only initiate allogeneic rejection of grafts, by either the direct or indirect pathway, but can also contribute to tolerance induction 1. DCs treated with a range of pharmacological inhibitors to prevent maturation and/or activation have been used to induce tolerance to alloantigens 1,2. The tolerogenic potential of DCs can be enhanced by genetic modification, including transfection/transduction with genes encoding immunomodulatory proteins or molecules that prevent DC activation 3,4.

We have developed two strategies for creating tolerogenic DCs. The first is to inhibit the expression of CD80/86 using a fusion protein, CTLA4-KDEL. The KDEL peptide retains/retrieves proteins to the ER 5. CTLA4-KDEL is therefore confined to the ER, where it binds CD80/86, preventing their passage to the cell surface. We have shown, using human cells, that inhibition of CD80/86 expression with CTLA4-KDEL results in DCs that can induce both anergy and regulatory activity in allospecific T cells 6.

The second strategy for creating tolerogenic DCs is to express IDO. IDO catabolises tryptophan, resulting in the production of kynurenines 7,8. Both the depletion of tryptophan and the production of kynurenines inhibit T-cell responses 9,10.

The aim of this study is to determine the ability of DCs, expressing either CTLA4-KDEL or IDO, to prevent corneal graft rejection. Cornea is the most commonly transplanted tissue 11, and while it is considered an immune-privileged tissue expressing various immunomodulatory enzymes such as IDO 12 and arginase 13, there is significant immunological rejection of corneal grafts with a 5-year graft survival of approximately 75% 14. As the cornea lacks resident DCs, rejection of the cornea occurs predominantly by the indirect pathway of allorecognition 15–18. In this study, we demonstrate long-term survival of corneal allografts after administering CTLA4-KDEL-expressing DCs targeted to the indirect pathway, and demonstrate the superiority of these cells over IDO-expressing DCs in preventing allograft rejection.

## Results

### Phenotype of transduced DCs

**Figure 1:**
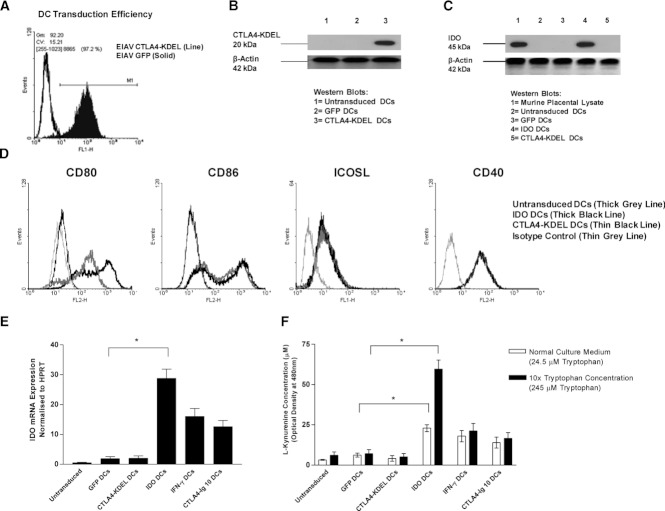
DC transduction with EIAV-CTLA4-KDEL or EIAV-IDO. BM-derived BALB/c DCs were transduced with EIAV on day 6 of culture prior to LPS stimulation on day 8. (A) DC transduction efficiency was assessed by GFP expression using flow cytometry 72 h after transduction with EIAV-GFP or EIAV-CTLA4-KDEL (control). Expression of the (B) 20 kDa CTLA4-KDEL protein and (C) 45 kDa IDO protein in DC lysates was determined by western blotting 72 h after transduction with EIAV-CTLA-KDEL or EIAV-IDO, respectively, and compared with expression in EIAV-GFP-transduced or untransduced DCs and, in the case of IDO, murine placenta (positive control). Expression of the 42 kDa β-actin housekeeping protein was measured as a loading control. (D) Flow cytometry histograms show the surface expression of CD80, CD86, ICOSL, and CD40 on untransduced DCs, and DCs 72 h after transduction with EIAV-CTLA4-KDEL or EIAV-IDO (control). The results shown in (A–D) are representative of three independent experiments. (E–F) Untransduced, immature DCs were treated on day 7 with either IFN-γ or CTLA4-Ig. The DC culture media was supplemented with L-tryptophan on day 6 (final concentration, 245 μM), followed by LPS stimulation on day 8. (E) DCs were harvested on day 9 for quantitative PCR analysis to assess IDO mRNA expression. (F) IDO activity was assessed by a kynurenine assay using DC culture supernatants that were either supplemented with tryptophan or unsupplemented. Results are shown as the mean ± SD of triplicate wells and are representative of three independent experiments performed. * *p* < 0.05, two-tailed *t-*test.

Lentiviral (equine infectious anaemia virus (EIAV)) constructs were generated encoding CTLA4-KDEL or murine IDO1. BALB/c DCs were transduced on day 6 of culture with EIAV-CTLA4-KDEL, EIAV-IDO, or EIAV-GFP (a control vector) followed by stimulation on day 8 with LPS. Transduction with EIAV-GFP resulted in more than 90% of DCs expressing GFP (Fig. 1A). Western blotting with anti-myc (CTLA4-KDEL contains a myc tag) (Fig. 1B) indicated that cells transduced with EIAV-CTLA4-KDEL expressed a protein of the expected size (∼20 kDa), while probing with anti-IDO indicated that cells transduced with EIAV-IDO expressed IDO (∼45 kDa) (Fig. 1C).

Transduced DCs, stimulated with LPS, were analysed for the expression of CD80, CD86, ICOSL and CD40. Cells transduced with EIAV-IDO showed a slight upregulation of expression of CD80 and possibly CD86 (Fig. 1D), consistent with non-specific activation of DCs following lentiviral transduction 19. Transduction with EIAV-CTLA4-KDEL resulted in virtual abolition of surface expression of CD80 and CD86, with no effect on the expression of ICOSL and CD40.

To compare the expression of IDO in DCs, cells were transduced with the lentiviral constructs, and the expression of IDO determined by RT-PCR and functional assays. As positive controls, DCs were treated with 10 μg/mL CTLA4-Ig or 60 ng/mL IFN-γ 20. EIAV-IDO resulted in upregulation of IDO expression, as determined by RT-PCR, to levels that were approximately twice that seen with IFN-γ or CTLA4-Ig treatment (Fig. 1E). The IDO was functional, as determined by production of kynurenines (Fig. 1F). No IDO upregulation was seen in EIAV-GFP or EIAV-CTLA4-KDEL-transduced DCs.

### Inhibition of allogeneic T-cell proliferation

**Figure 2:**
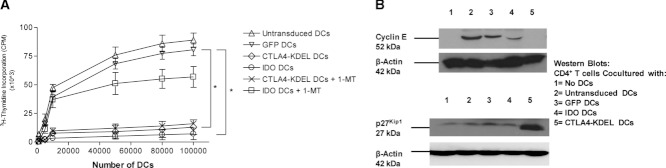
Inhibition of allogeneic T-cell proliferation after DC expression of CTLA4-KDEL or IDO. BALB/c (H-2^d^) DCs were transduced on day 6 of culture with EIAV-GFP (control), EIAV-CTLA4-KDEL or EIAV-IDO and stimulated with LPS on day 8 of culture. All DC populations were cultured on day 9 with fully MHC-disparate, spleen-derived C3H/He CD4^+^ T cells. Where indicated, 250 μM 1-methyl-D,L-tryptophan (1-MT), in combination with excess tryptophan, was added to the medium at the start of coculture. (A) Increasing numbers of both EIAV transduced and untransduced DC populations (0–10^5^) were co-cultured with C3H/He-derived CD4^+^ T cells. Proliferation of CD4^+^ T cells was detected by thymidine incorporation on day 5 of the MLR, and the results are shown as the mean ± SD of triplicate wells and are representative of three independent experiments. *p < 0.05, two-tailed *t-*test. (B) Expression of the 52 kDa cyclin E protein and p27^Kip1^ protein (and the 42 kDa β-actin protein), in the lysates of CD4^+^ T cells incubated with either the transduced or untransduced DCs described was determined by western blotting on day 4 of co-culture. Data shown are representative of three independent experiments performed.

To assess the functional effect of CTLA4-KDEL and IDO expression on the ability of DCs to stimulate allogeneic T cells, BALB/c DCs were transduced, activated with LPS and then used to stimulate fully MHC-disparate C3H/He CD4^+^ T cells. DCs transduced with EIAV-CTLA4-KDEL and EIAV-IDO did not stimulate allogeneic T-cell proliferation (Fig. 2A). Addition of the IDO inhibitor 1-methyl tryptophan largely restored the ability of EIAV-IDO, but not EIAV-CTLA4-KDEL, transduced DCs to stimulate T cells.

To further determine the effect of DC expression of CTLA4-KDEL or IDO on the T cells, the expression of cyclin E (a marker of cell division 21) and p27^Kip1^ (associated with anergic T cells 22) by allogeneic T cells, was determined. Minimal p27^Kip1^ expression is seen in resting T cells or T cells incubated with untransduced or EIAV-GFP-transduced DCs (Fig. 2B), while incubation with untransduced or EIAV-GFP-transduced DCs resulted in cyclin E induction. T cells incubated with CTLA4-KDEL-expressing DCs show no increase in cyclin E, but a rise in p27^Kip1^. The effect of EIAV-IDO-transduced DCs is intermediate with a slight rise in cyclin E and no significant upregulation of p27^Kip1^.

### Induction of T-cell anergy and regulation in vitro

Rechallenge MLRs were used to determine whether CTLA4-KDEL- or IDO-expressing DCs were capable of inducing anergy in allogeneic CD4^+^ T cells. The fully mismatched BALB/c–C3H combination described above results in direct pathway alloantigen presentation 23,24. As the indirect pathway is important in corneal rejection, we used a CBK (stimulator) to CBA (responder) combination. The CBK mouse is transgenic for K^b^ on a CBA (H-2^k^) background 25, so presentation of alloantigen to CBA CD4^+^ T cells would occur obligatorily by the indirect pathway 26,27. For third-party controls, we used DCs derived from B10.A-H-2^a^ mice, congenic for class I D^d^, so again resulting in indirect alloantigen presentation to CBA CD4^+^ T cells. EIAV transduced (or control) CBK DCs were used to stimulate CBA CD4^+^ T cells in the first stage of the MLR. After 10 days, the T cells were removed and rechallenged with either CBK DCs or B10.A DCs. Proliferation was determined on days 3, 5 and 7.

**Figure 3:**
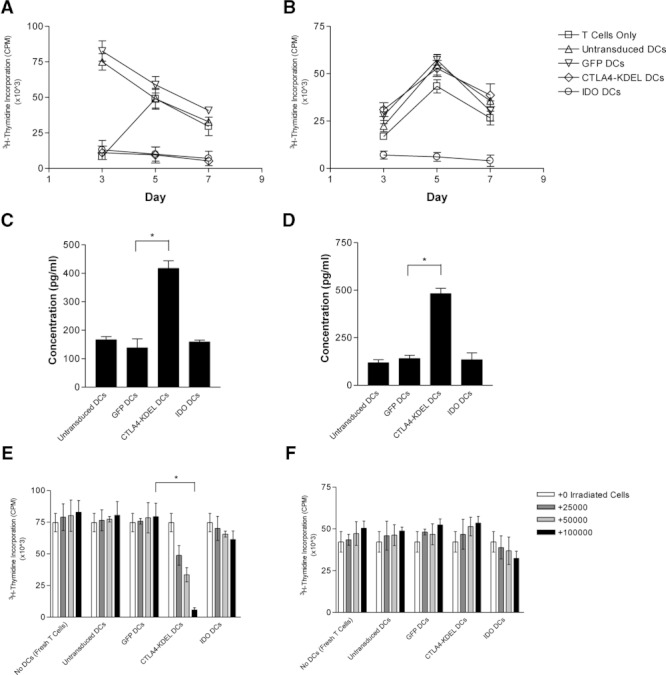
Induction of T-cell anergy and regulation in vitro with indirect donor allospecificity. CBK (H-2^k^ + K^b^) DCs were transduced with either EIAV-GFP (control), EIAV-CTLA4-KDEL or EIAV-IDO, followed by stimulation with LPS. The transduced (and untransduced) DCs were subsequently incubated in vitro with CBA/Ca-derived CD4^+^ T cells. After 10 days, T cells were harvested and rechallenged in vitro with (A) donor CBK DCs or (B) third-party B10.A (H-2^k^ + D^d^) DCs, and CD4^+^ T-cell proliferation was assessed by thymidine incorporation on days 3, 5 and 7. (C–D) Culture supernatant from the donor DC rechallenge assay was harvested for detection of the immunoregulatory cytokines (C) TGF-β and (D) IL-10 by ELISA. (E–F) In addition, after 10 days of the primary DC-CD4^+^ T-cell coculture, T cells were also harvested, irradiated and added (0–10^5^ CD4^+^ T cells) to a primary MLR between freshly isolated CBA CD4^+^ T cells and (E) donor CBK DCs or (F) third-party B10.A DCs. T-cell proliferation was assessed by thymidine incorporation after 5 days. All results are shown as the mean ± SD of triplicate wells and are representative of three independent experiments performed. *p < 0.05, two-tailed *t*-test.

Fresh T cells (not exposed to a primary culture) showed maximal proliferation on day 5. However, T cells that had been previously incubated with untransduced or EIAV-GFP-transduced CBK DCs showed maximal proliferation on day 3, consistent with prior exposure to alloantigen. T cells that had been previously exposed to CTLA4-KDEL- or IDO-expressing CBK DCs showed minimal proliferation at all time points to CBK DCs (Fig. 3A). Following rechallenge with third-party B10.A DCs, T cells previously exposed to control DCs showed maximal proliferation on day 5 (Fig. 3B). T cells previously incubated with CTLA4-KDEL-expressing DCs showed similar proliferation, indicating that the lack of proliferation seen to CBK DCs was alloantigen-specific. However, T cells exposed to IDO-expressing DCs showed little proliferation at any time point, indicating that the suppression was not alloantigen specific. Increased levels of both TGF-β (Fig. 3C) and IL-10 (Fig. 3D) were seen in supernatants from T cells rechallenged with CBK DCs following primary incubation with CTLA4-KDEL-expressing CBK DCs, but not IDO-expressing or control DCs.

To determine whether indirect-specific T cells exposed to CTLA4-KDEL- or IDO-expressing DCs are capable of regulating other T cells, we incubated CBA CD4^+^ T cells with virally transduced (or control) CBK DCs for 10 days. T cells were recovered, irradiated and then added back to a culture containing naïve CBA T cells and either CBK or third-party B10.A DCs. T cells that had been exposed to CTLA4-KDEL-expressing DCs inhibited the response of fresh T cells to CBK DCs (Fig. 3E) but not third-party DCs (Fig. 3F), demonstrating T-cell regulation with indirect pathway specificity. No inhibition was seen with T cells exposed to control or IDO-expressing DCs (Fig. 3E and F). There was no induction of FoxP3 expression in T cells in vitro. While increased IL-10 and TGF-β was seen in supernatant from the cultures previously exposed to CTLA4-KDEL-expressing CBK DCs, no such increase was seen in suppressor assays containing third-party DCs (data not shown).

Alloantigen-specific induction of anergy was also observed using a direct pathway (multiple mismatch) strain combination with CTLA4-KDEL-expressing BALB/c DCs and C3H CD4^+^ T cells, and associated with secretion of TGF-β and IL-10, while expression of IDO by BALB/c DCs resulted in inhibition of both specific and third-party alloresponses (Supporting Information Fig. 1A–D). C3H T cells that had been exposed to CTLA4-KDEL-expressing BALB/c DCs also inhibited the response of fresh T cells to BALB/c but not third-party DCs (Supporting Information Fig. 1E and F). Only a moderate, but significant, inhibition was seen with T cells exposed to IDO-expressing DCs.

### Induction of T-cell anergy in vivo

**Figure 4:**
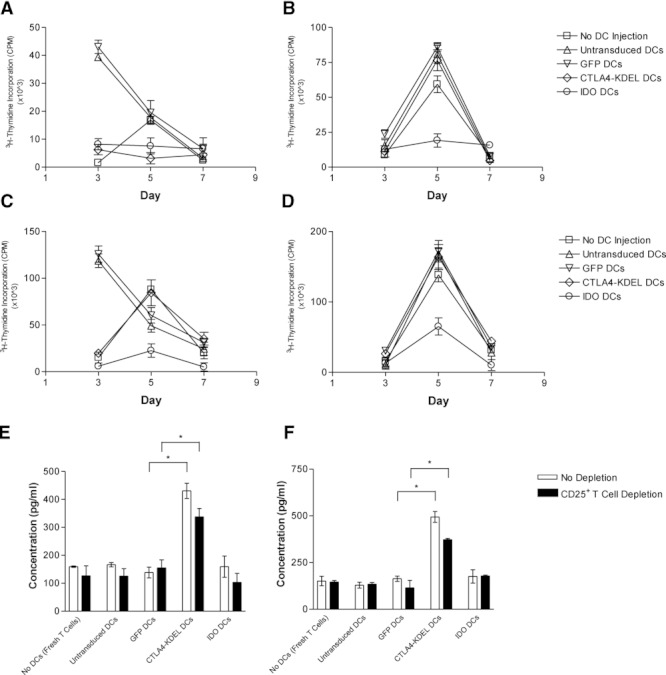
Induction of T-cell anergy in vivo with indirect donor allospecificity and production of immunoregulatory cytokines. 2.5 × 10^6^ CBK DCs (either untransduced, or transduced with EIAV-GFP, EIAV-CTLA4-KDEL or EIAV-IDO) were injected i.v. into CBA/Ca mice. (A–D) After 10 days, CD4^+^ T cells purified from the spleens of injected mice were rechallenged in vitro with (A) donor CBK DCs or (B) third-party B10.A DCs, and CD4^+^ T-cell proliferation was assessed by thymidine incorporation on days 3, 5 and 7. The purified CD4^+^ T cells were also rechallenged with (C) donor CBK DCs or (D) third-party B10.A DCs in the presence of 100 U/mL exogenous IL-2. (E–F) Culture supernatant from donor DC rechallenge assays (including those from CD4^+^CD25^+^ T-cell-depleted rechallenge assays) was harvested for detection of the immunoregulatory cytokines (E) TGF-β and (F) IL-10 by ELISA. All results are shown as the mean ± SD of triplicate wells and are representative of three independent experiments performed. *p < 0.05, two-tailed *t*-test.

To determine that if modified DCs can induce anergy with indirect donor allospecificity in vivo, CD4^+^ T cells, purified from the spleens of CBA mice 10 days after CBK DC administration, were challenged in vitro with CBK or third-party B10.A DCs. T-cell proliferation was determined on days 3, 5 and 7. CD4^+^ T cells from mice given control DCs showed a rapid response to CBK DCs, while T cells from naïve animals showed a maximal response on day 5 (Fig. 4A). Mice given DCs expressing either CTLA4-KDEL or IDO showed minimal T-cell proliferation. The response of T cells to third-party DCs was normal in all cases, except following injection of IDO-expressing DCs in which a reduced response was seen (Fig. 4B). Addition of IL-2 (100 U/mL) to the cultures restored the ability of T cells from mice given CTLA4-KDEL-expressing DCs, but not IDO-expressing DCs, to respond to CBK DCs (Fig. 4C and D). Similar results were obtained following the injection of modified BALB/c DCs into C3H mice (Supporting Information Fig. 2A–D). These data indicate that CTLA4-KDEL-expressing DCs can induce anergy in alloantigen-specific T cells in vivo*,* but IDO-expressing DCs induce generalised unresponsiveness. The alloantigen-specific unresponsiveness in T cells exposed to CTLA4-KDEL-modified DCs is associated with increased concentrations of IL-10 and TGF-β on day 7 of the culture for both the CBK-CBA combination (Fig. 4E and F), and the BALB/c-C3H combination (Supporting Information Fig. 2E and F). This was seen both with CD4^+^ T cells and CD4^+^ T cells depleted of CD25 cells.

### CTLA4-KDEL-expressing DCs can induce Treg cells in vivo

**Figure 5:**
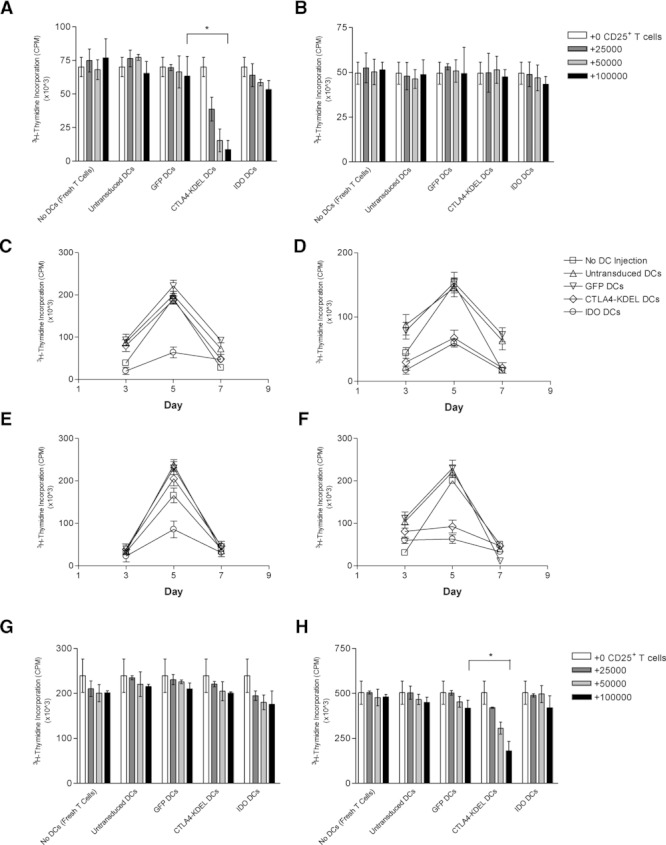
Generation of Treg cells in vivo with indirect donor allospecificity and capacity for linked suppression. 2.5 × 10^6^ CBK DCs (either untransduced, or transduced with EIAV-GFP (control), EIAV-CTLA4-KDEL or EIAV-IDO) were injected i.v. into C3H/He mice. (A–B) After 10 days, CD4^+^CD25^+^ T cells purified from the spleens were irradiated and added (0–10^5^ CD4^+^CD25^+^ T cells) to a primary MLR between freshly isolated CBA/Ca-derived CD4^+^ T cells and (A) donor CBK DCs or (B) third-party B10.A DCs. T-cell proliferation was assessed by thymidine incorporation after 5 days. (C–D) CD4^+^ T cells purified from the spleens of injected mice were also rechallenged in vitro with (C) (BALB/c × C57BL/6)F1 DCs expressing only third-party MHC or (D) (C57BL/6 × CBA/Ca)F1 DCs expressing both donor (K^b^ presented in the context of I-A^k^/I-E^k^) and third-party MHC, and CD4^+^ T-cell proliferation was assessed by thymidine incorporation on days 3, 5 and 7. (E–F) Rechallenge assays were repeated using CD4^+^ T cells that were depleted of CD4^+^CD25^+^ T cells prior to rechallenge with (E) (BALB/c × C57BL/6)F1 DCs or (F) (C57BL/6 × CBA/Ca)F1 DCs. (G–H) In addition, CD4^+^CD25^+^ T cells purified from the spleens of injected mice were irradiated and added (0–10^5^ CD4^+^CD25^+^ T cells) to new primary MLRs between freshly isolated CBA/Ca-derived CD4^+^ T cells and (G) (BALB/c × C57BL/6)F1 DCs or (H) (C57BL/6 × CBA/Ca)F1 DCs and T-cell proliferation assessed by thymidine incorporation after 5 days. All results are shown as the mean ± SD of triplicate wells and are representative of three independent experiments performed. *p < 0.05, two-tailed *t*-test.

To determine if administration of CTLA4-KDEL- or IDO-expressing DCs can induce Treg cell activity in vivo, 2.5 × 10^6^ CBK DCs (virally transduced or control) were injected i.v. into CBK mice. After 10 days, CD4^+^CD25^+^ splenocytes were purified, irradiated and then added to a fresh culture of CBA CD4^+^ T cells and CBK DCs and the proliferation assessed on day 5. Addition of T cells from mice given CTLA4-KDEL-expressing DCs, but not from mice given IDO-expressing- or control DCs, inhibited allogeneic proliferation of fresh CBA T cells (Fig. 5). No inhibition was observed when B10.A DCs were used as stimulators (Fig. 5B). Similar induction of alloantigen-specific Treg cells was seen following the administration of CTLA4-KDEL-expressing BALB/c DCs to C3H animals, with no evidence of Treg cells in mice given IDO-expressing DCs (Supporting Information Fig. 3A and B). As shown in Supporting Information Fig. 4, there was no increase in FoxP3 expression (∼6%) in C3H animals given untransduced, GFP- or IDO-transduced BALB/c DCs, but FoxP3 expression increased (∼20%) in mice given CTLA4-KDEL-expressing DCs.

To determine if the indirect pathway-specific regulatory cells can mediate linked suppression, CD4^+^ T cells were isolated from CBA mice given unmodified or virally transduced CBK DCs. These were then stimulated with (C57BL/6 × CBA)F1 DCs (capable of presenting K^b^ in the context of H-2^k^, as well as H-2^b^ directly) or (BALB/c × C57BL/6)F1 DCs (incapable of presenting K^b^ in the context of H-2^k^), and proliferation determined on days 3, 5 and 7 (Fig. 5C and D). We have previously shown that T cells exposed to CTLA4-KDEL-expressing CBK DCs in vivo will not subsequently respond to CBK DCs but will show a normal naïve response to B10.A DCs (Fig. 4A and B). However, they do not respond to (C57BL/6 × CBA)F1 DCs, indicating that linked suppression occurred when novel H-2^b^ alloantigens were presented on the same DC as K^b^ in the context of H-2^k^. The response to the control (BALB/c × C57BL/6)F1 DCs (where K^b^ is not presented in the context of H-2^k^) was normal. T cells exposed to IDO-expressing DCs showed a generalised lack of response to all alloantigens. The linked suppression mediated by T cells, exposed in vivo to CTLA4-KDEL-expressing CBK DCs, was also observed in rechallenge cultures that were depleted of CD4^+^CD25^+^ T cells (Fig. 5E and F), indicating (together with ELISA results shown previously (Fig. 4E and F)) that CD4^+^CD25^−^ T cells, in addition to CD4^+^CD25^+^ T cells, have a role in indirect pathway-specific T-cell regulation.

T cells exposed to CTLA4-KDEL-expressing DCs in vivo also mediated linked suppression of fresh T cells, as shown by addition of irradiated CD4^+^CD25^+^ CBA T cells, from animals given virally transduced or control CBK DCs, to cultures in which (C57BL/6 × CBA)F1 or (BALB/c × C57BL/6)F1 DCs were used to stimulate fresh CBA T cells (Fig. 5G and H). T cells exposed to CTLA4-KDEL-expressing DCs in vivo, but not to other DCs, suppressed the response to (C57BL/6 × CBA)F1 DCs but not (BALB/c × C57BL/6)F1 DCs. Similar linked suppression and regulation was also seen in C3H mice given fully mismatched BALB/c CTLA4-KDEL-expressing DCs (data not shown).

### Enhanced survival of EIAV-transduced DCs in vivo

**Figure 6:**
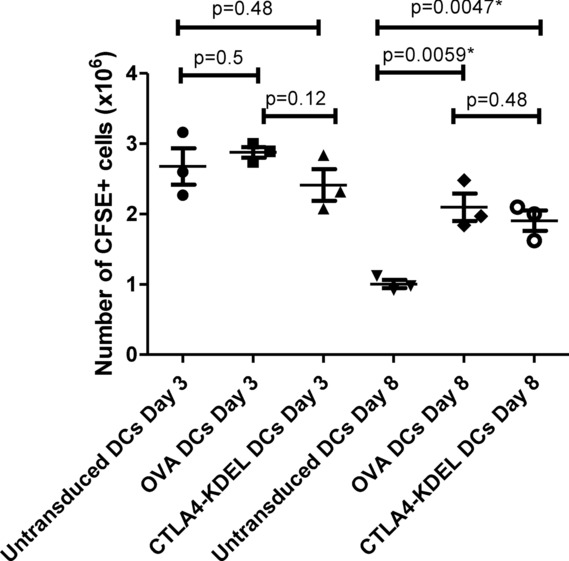
Enhanced survival of equine infectious anaemia virus (EIAV)-transduced DCs in vivo. 1 × 10^7^ CFSE-labelled C3H DCs (either untransduced, or transduced with EIAV CTLA4-KDEL or EIAV OVA) were injected intravenously into female syngeneic mice. The spleens from these mice were harvested on days 3 and 8 post-injection and the total number of CFSE-labelled DCs present assessed by flow cytometric analysis. Each symbol represents an individual animal and bars represent the mean ± SD; data shown are representative of three independent experiments performed. *p < 0.05, two-tailed *t*-test.

In other models, the administration of tolerogenic DCs has led to immunisation of the recipient due to re-presentation of antigen, obtained from dead donor DCs, by host DCs 28. However, reports have indicated that lentiviral-transduced DCs have an increased survival in vivo, which would reduce such re-presentation 29. In order to investigate the survival of EIAV-transduced DCs in vivo, CFSE-labelled DCs (either untransduced, or transduced with EIAV CTLA4-KDEL or EIAV-OVA) were injected into syngeneic mice. The number of CFSE^+^ DCs in the spleens was determined on days 3 and 8 post-injection. Transduction with either EIAV vector led to increased survival of DCs on day 8 (Fig. 6).

### Prolongation of corneal graft survival following administration of modified DCs

**Figure 7:**
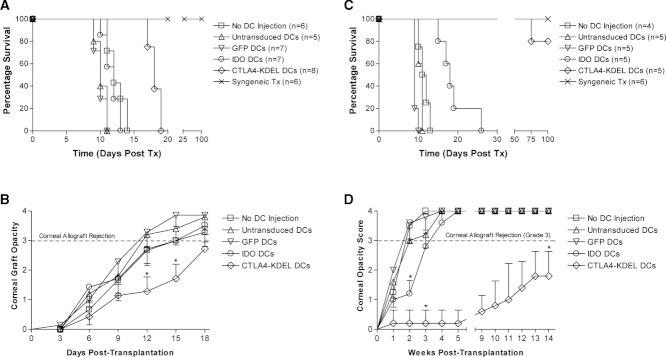
Prolongation of corneal graft survival following administration of modified DCs. BALB/c mice were either untreated (*n* = 6) or given 2.5 × 10^6^ C3H/He DCs intravenously. The DCs were either untransduced (*n* = 5), or transduced 72 h earlier ex vivo with equine infectious anaemia virus (EIAV)-GFP (control, *n* = 7), EIAV-IDO (*n* = 7) or EIAV-CTLA4-KDEL (*n* = 8). Ten days later, the BALB/c mice received a complete MHC-disparate C3H/He corneal graft or a syngeneic BALB/c graft (*n* = 6). (A) Data were plotted using the Kaplan–Meyer method and differences in graft survival were analysed using a log-rank test. (B) Corneal graft opacity scores were plotted and statistical differences between CTLA4-KDEL- and GFP (control)-expressing DCs calculated using the Mann–Whitney *U* test. * *p* < 0.008. (C) Using the same protocol, BALB/c mice were either untreated (*n* = 4) or given 2.5 × 10^6^ (CBA × BALB/c)F1 DCs intravenously. The DCs were either untransduced (*n* = 5), or transduced 72 h earlier ex vivo with EIAV-GFP (*n* = 5), EIAV-IDO (*n* = 5) or EIAV-CTLA4-KDEL (*n* = 5). 10 days later, BALB/c mice received a complete MHC-disparate CBA/Ca corneal graft or a syngeneic BALB/c graft (*n* = 6). Data were analysed as above. (D) Corneal graft opacity scores were plotted and statistical differences between CTLA4-KDEL- or IDO-expressing DCs and GFP-expressing DCs calculated using the Mann–Whitney *U* Test. *p < 0.008. Data shown are representative of three independent experiments performed.

A mouse model was used to determine if corneal graft survival could be prolonged by the administration of allogeneic CTLA4-KDEL- or IDO-expressing DCs. As there is a high rate of graft failure in agouti mice as a result of glaucoma 30,31, we used a fully MHC-disparate C3H (H-2^k^) → BALB/c (H-2^d^) combination. Unmodified or virally transduced C3H DCs (2.5 × 10^6^) were administered i.v. 10 days prior to transplantation. Administration of CTLA4-KDEL-expressing DCs resulted in moderate prolongation of graft survival (median survival time: 18 days) compared with that of GFP-transduced DCs (10 days), unmodified DCs (11 days), IDO-transduced DCs (12 days) or naive animals (12 days) (Fig. 7A). Similar results are seen when measuring the development of corneal opacity with a slight reduction in the kinetics of rejection in recipients of CTLA4-KDEL-expressing DCs (Fig. 7B).

The use of donor-type DCs would favour tolerance induction to the direct pathway alone. However, corneal graft rejection is predominantly by the indirect pathway. We therefore pre-treated BALB/c mice with unmodified or virally transduced (CBA/Ca × BALB/c)F1 DCs 10 days prior to a CBA (H-2^k^) graft. The use of F1 DCs permits presentation of alloantigen by both the indirect and direct pathway. There was considerable prolongation of graft survival in animals receiving CTLA4-KDEL-expressing DCs (median survival time > 100 days), when compared with that of animals treated with no DCs (12 days), unmodified DCs (11 days) or control GFP-transduced DCs (9 days) (Fig. 7C). Pre-treatment with IDO-expressing F1 DCs resulted in enhanced graft survival (18 days) compared with that of controls. Post-graft opacity scores show that recipients of CTLA4-KDEL F1 DCs showed complete graft transparency up to 75 days post graft (Fig. 7D). These data indicate that F1 DCs expressing CTLA4-KDEL are capable of profoundly influencing graft survival, while IDO-expressing DCs have a minor effect.

## Discussion

In this study we have investigated two potential DC-based strategies for tolerance induction; the intracellular retention of CD80/86 by CTLA4-KDEL and the expression of IDO. We transduced DCs using lentiviruses. One disadvantage of this (as previously observed with human DCs 19, and seen in this study) is that it can lead to maturation of DCs. However, this may improve the efficiency with which the cells traffic to appropriate sites for tolerance induction 32.

We have demonstrated that CTLA4-KDEL- and IDO-expressing DCs are unable to induce and/or sustain allogeneic T-cell proliferation. In addition, as previously seen using human cells, exposure of allogeneic T cells to CTLA4-KDEL-expressing DCs rendered alloantigen-specific T cells anergic, and generated a population of Treg cells. This was seen both in vitro and upon in vivo challenge with transduced DCs. CTLA4-KDEL-expressing DCs were capable of modulating both direct and indirect pathway allogeneic responses and were capable of cross regulation. The induction of regulatory cell activity by CTLA4-KDEL-expressing DCs was associated with an increase in FoxP3^+^CD4^+^CD25^+^ T cells in vivo. Furthermore, regulatory cells were induced in both CD25^+^ and CD25^−^ T-cell compartments. The induction of regulatory cells was associated with the production of IL-10 and TGF-β.

In other experimental systems, administration of DCs with tolerogenic potential has led to the induction of indirect pathway allospecific T cells due to re-presentation of alloantigen derived from dead donor DCs, leading to graft rejection 28. However, lentiviral transduction can increase in vivo DC survival 29. We observed an increase in survival of DCs transduced with EIAV. This would reduce re-presentation and the induction of an indirect alloresponse by this pathway. This emphasises the importance of factors such as DC survival in determining the tolerogenic ability of DCs.

In contrast, we have shown that IDO-expressing DCs induced a generalised T-cell hyporesponsiveness, both in vitro and in vivo, and there was no significant induction of anergic or Treg cells. This is in contrast to in vitro data we have reported with IDO-expressing human DCs in which donor-specific anergy was induced, albeit with a significant level of T-cell death 33. Although some groups have reported the induction of Treg cells by IDO-expressing DCs 34–36, others have failed to see any such induction 37. It is possible that the effect of IDO can vary, with low levels inducing antigen-specific anergy and regulation, and higher levels resulting in non-specific hyporesponsiveness and death.

We used a corneal transplant model to determine whether either CTLA4-KDEL- or IDO-expressing DCs could prolong graft survival. Corneal graft rejection occurs primarily through the indirect pathway of allorecognition due to the paucity of DCs in the cornea 15–17. MHC class II^−^ DCs have been observed in the cornea, though the function of these cells remain unclear 38. MHC class II^+^ donor derived DCs have been demonstrated in cervical lymph nodes draining corneal allografts 39, and could initiate direct pathway alloreactivity.

We showed that if donor-origin DCs were administered to mice prior to transplantation, CTLA4-KDEL-expressing cells caused a moderate prolongation of graft survival. These cells would be capable of presenting alloantigen by the direct pathway alone, and so extended survival indicates either (i) non-specific immunosuppression or (ii) that inhibition of the direct pathway can affect graft survival. However, (donor × recipient)F1 DCs, which present by the direct and indirect pathways, resulted in long-term graft survival. These data highlight the role of the indirect pathway in corneal graft rejection. It is noteworthy that prolongation of graft survival was seen in the absence of any other treatment. Previously, when dexamethasone-treated F1 DCs were injected into rats to induce indirect allospecific tolerance, the administration of a single dose of CTLA4-Ig was necessary to prevent sensitisation caused by representation of donor DC-derived alloantigens 2.

In contrast to CTLA4-KDEL, IDO-expressing DCs were less effective, with only a moderate increase in survival using F1 DCs. Previously, we have shown that IDO expression in the graft itself can prolong corneal graft survival 11. However, in such a setting, IDO operates to block the effector cell response, rather than inhibit alloreactive T-cell activation. It may be that IDO has more potential to protect tissues from damage than to prevent T-cell activation. We have also shown, however, that topical and systemic administration of kynurenines suppresses CD4^+^ T-cell proliferation and prolongs corneal allograft survival 40.

In these experiments we have used F1 DCs to present alloantigen by the indirect pathway, which is not clinically applicable. There are several alternatives that might be feasible that include pulsing recipient DCs with alloantigen-derived protein or peptide. However, in order to achieve long-term presentation by the indirect pathway, we favour further genetic modification of the DCs to express donor-type alloantigen. This approach has the advantage over administration of CTLA4-Ig in being both alloantigen specific and not causing upregulation of IDO in DCs.

Whilst these present studies are restricted to corneal grafts, the use of CTLA4-KDEL DCs could be important in other settings. Given the strength of the direct pathway after transplantation of vascularised organs, the most plausible strategy for inducing tolerance in the clinical setting is a dual approach in which the frequency of direct pathway alloreactive T cells is reduced by deletion and/or anergy, and tolerance of the residual direct and the indirect pathway then induced by a regulatory mechanism 41. Therefore, a combination of short-term immunosuppression during the acute phase of rejection post-transplantation and the administration of tolerogenic CTLA4-KDEL-transduced recipient DCs, presenting antigen by the indirect pathway, prior to the chronic phase of allograft rejection may prove to be a clinically applicable strategy to achieve donor-specific transplantation tolerance.

## Materials and methods

### Mice

CBK mice 25 were bred in-house. All other mice were purchased from Harlan Olac (Bicester, UK). Animals were treated in accordance with UK regulations and the ARVO Statement for the Use of Animals in Ophthalmic and Vision Research.

### Generation of lentiviral constructs

The extracellular domain of murine CTLA4 was amplified from cDNA of activated T cells, inserted into the pCMV/*myc*/ER vector (Invitrogen, Paisley, UK) and then subcloned into the EIAV plasmid pSMART2G 42 (Oxford Biomedica Co., Oxford, UK) replacing the GFP gene, resulting in pSMART-CTLA4-KDEL. The pSMART-IDO construct encoding murine IDO1 has been previously described 12. The li-OVA gene, encoding the murine invariant chain (li) in which the CLIP has been replaced by the OVA peptide (OVA_328–339_), was generated by excision from the pSL8-lipOVA vector (donated by Dr. Stephen Thirdborough, Southampton University) and subcloned into the EIAV plasmid. pSMART2G, encoding GFP, was used as a reporter construct and control. Vesicular stomatitis virus G (VSV-G)-pseudotyped EIAV lentiviruses were produced using three-plasmid cotransfection of 293T cells followed by ultracentrifugation as described 19,43.

### Murine BM-derived DC cultures

DCs were generated from murine BM as described 44,45 and cultured in RPMI 1640 medium (Invitrogen) supplemented with 10% heat-inactivated foetal calf serum (PAA, Pasching, Austria), 100 units/mL penicillin, 100 μg/mL streptomycin, 2 mM L-glutamine (Cambrex Biosciences, Wokingham, UK) and 50 μM β-mercaptoethanol (Invitrogen) (complete medium) and supernatant from a GM-CSF-producing cell line 46. Where indicated, cells were treated with LPS, murine IFN-γ (PeproTech EC, London, UK) or mouse CTLA4-Ig (R&D Systems, Abingdon, UK). DCs were transduced with EIAV lentiviral vectors on day 6 of culture at MOI 300 for 72 h.

### Flow cytometry

Flow cytometry was carried out as previously described 19,47,48 using the following antibodies: ICOSL (GL1) (Insight Biotechnology, Wembley, UK); CD40 (3/23) (Serotec, Kidlington, UK), CD80 (RMMP-1) and CD86 (RMMP-2) (Caltag, Buckingham, UK). Purified Rat IgG2a (54447) antibody was used as an isotype control. Goat anti-rat IgG-PE (BD Biosciences, Oxford, UK) was used as a secondary antibody. FoxP3-allophycocyanin (FJK-16s) and rat IgG2a-allophycocyanin (eBioscience, USA) were used for intracellular detection of FoxP3.

### Immunoblotting

Cells (1–2 × 10^6^) were resuspended in 130 μL lysis buffer (1% NP-40, 150 mM NaCl, 5 mM MgCl_2_, and 10 mM Hepes buffer) supplemented with Protease Inhibitor Cocktail (Sigma-Aldrich, Poole, UK) and incubated on ice for 30 min followed by centrifugation at 4000 × *g* for 5 min. The lysate was separated by SDS-PAGE under reducing conditions and transferred to a nitrocellulose membrane. Membranes were probed using antibodies specific for IDO (rabbit polyclonal IgG, Cosmo Bio Co. Ltd, Japan); *c-myc* epitope tag (4A6) and cyclin E (rabbit polyclonal IgG) (Upstate-Millipore, Watford, UK); p27^Kip1^ (G173–524, BD Biosciences); β-actin (AC-15, Sigma-Aldrich). The following secondary antibodies were used: goat anti-rabbit IgG HRP, rabbit anti-mouse IgG HRP and rabbit anti-goat IgG HRP (Dako-Millipore). Proteins were visualised using the ECL™ plus Western Blotting detection system (Amersham Biosciences, Little Chalfont, UK).

### T-cell purification

Splenocytes were obtained by passing a spleen through 70 μm cell strainers into cold-complete medium with 10 units/mL DNAse-Pulmozyme/Dornase Alfa (Roche Applied Sciences, Lewes, UK). Following lysis of erythrocytes, splenocytes were incubated on a horizontal roller at 4°C with rat antibodies specific for CD45R/B220 (RA3-3A1), CD8 (53.6.7), H2-E^k,d^/A^b,d^ (M5/114.15.2), and CD16/32 (2.4G2 all ATCC Manassas, VA, USA), washed twice in RPMI 1640 medium and incubated with sheep anti-rat IgG Dynabeads® (Dynal Biotech, Bromborough, UK) on a horizontal roller at 4°C before magnetic separation to obtain CD4^+^ T cells. Where indicated, CD25^+^ T cells were purified or depleted from CD4^+^ T-cell populations using the MACS® CD25 MicroBead Kit (Miltenyi Biotec GmbH, Bergisch Gladbach, Germany).

### T-cell proliferation assays

CD4^+^ T cells were cultured for 5 days with allogeneic BM-derived DCs in complete medium. Unless otherwise stated, 25 × 10^4^ CD4^+^ T cells to 5 × 10^4^ DCs were used. Cells were pulsed on day 4 with ^3^H-thymidine (1 μCi/well) (Amersham Biosciences) and harvested 17 h later. Two-stage MLRs (rechallenge assays) were performed using modifications of established protocols 6,49. To investigate the induction of anergy and linked suppression in vitro, DC populations were incubated with allogeneic CD4^+^ T cells (2 × 10^5^ DCs:2 × 10^6^ T cells) for 10 days. The T cells were harvested, washed twice in PBS and rechallenged with WT DCs (1 × 10^4^ DCs:2 × 10^4^ T cells). To investigate the induction of anergy and linked suppression in vivo, 2.5 × 10^6^ DCs were injected i.v. via the tail vein. Ten days later, CD4^+^ splenocytes were purified and rechallenged with DCs as described for the assays to investigate anergy induction and linked suppression in vitro. Where indicated 100 U/mL exogenous IL-2 (Roche) was added to cultures. Proliferation in rechallenge experiments was determined by thymidine incorporation on days 3, 5 and 7. Cytokines were detected in culture supernatants using mouse IL-10 and TGF-β1 ELISA Kits (eBioscience). To determine functional Treg-cell activity, CD4^+^ T cells were harvested on day 10 of primary MLRs. Alternatively, CD4^+^CD25^+^ T cells were purified from the spleens and lymph nodes of mice following injection with DCs 10 days previously. The cells were irradiated (50 Gy) and added (0–100 000 cells) to ‘fresh’ MLRs, consisting of WT DCs and allogeneic CD4^+^ T cells.

### CFSE labelling

BM-derived C3H DCs, either untransduced or transduced with EIAV-CTLA4-KDEL or EIAV-OVA, were labelled with CFSE using the Vybrant® CFDA SE Cell Tracer Kit (Molecular Probes-Invitrogen, Paisley, UK). Female C3H mice were injected with 1 × 10^7^ CFSE-labelled syngeneic DCs. Spleens were harvested on days 3 and 8 post-injection. CFSE-labelled DCs present in the spleen were detected using a FACScalibur flow cytometre. Fifty thousand events were collected per mouse and the percentage of FL1-positive cells used to calculate the total number of CFSE-labelled DCs present per spleen.

### Quantitative PCR

Messenger RNA was prepared using TRIzol™ (Invitrogen) and reverse transcribed using M-MLV RT (Promega), according to the manufacturers’ instructions. PCR was performed using a LightCycler (Roche Molecular Biochemicals, Hertfordshire, UK) and SYBR® Green Taq ReadyMix™ (Sigma-Aldrich) according to the manufacturers’ instructions. The programme was 95°C for 3 min followed by 40 cycles of (95°C for 5 s, 56°C for 10 s, and 72°C for 13 s) followed by quantification at 81°C. The IDO primers were 5′-TGGCAAACTGGAAGAAAAAG-3′ (forward) and 5′-AATGCTTTCAGGTCTTGACG-3′ (reverse). Hypoxanthine phosphoribosyl transferase primers were 3′-GTAATGATCCAGTCAACGGGGGAC-5′ (forward) and 3′-CCAGCAAGCTTGCAACCTTAACCA-5′ (reverse) 11.

### L-kynurenine assay

L-kynurenine was measured as described 12,50,51.

### Orthotopic corneal transplantation and criteria for graft rejection

Murine corneal transplantation was performed in the right eye as described 52,53. The eyes were examined every 2 days following suture removal on day 7. The grading of corneal opacity and the onset of graft rejection were graded as described by an examiner masked to the treatment group 53.

## Abbreviations

EIAV: equine infectious anaemia virus
HPRT: hypoxanthine phosphoribosyl transferase
MST: median survival time
1-MT: 1-methyl tryptophan
VSV-G: vesicular stomatitis virus G

## Supplementary material

As a service to our authors and readers, this journal provides supporting information supplied by the authors. Such materials are peer reviewed and may be re-organized for online delivery, but are not copy-edited or typeset. Technical support issues arising from supporting information (other than missing files) should be addressed to the authors.
