# Supplementary material for: Dendritic cell modification as a route to inhibiting corneal graft rejection by the indirect pathway of allorecognition
Source: Eur J Immunol. 2012 Dec 4;43(3):734–46. doi: 10.1002/eji.201242914 (PMC3615172; doi:10.1002/eji.201242914)
Supplement: Supplementary file 1 [file eji0043-0734-SD1.pdf]

European Journal of Immunology

Supporting Information
for
DOI 10.1002/eji.201242914

Adnan Khan, Hongmei Fu, Lee Aun Tan, Jennifer E. Harper, Sven C. Beutelspacher, Daniel F. P. Larkin, Giovanna Lombardi, Myra O. McClure and Andrew J. T. George

Dendritic cell modification as a route to inhibiting corneal graft rejection by the indirect pathway of allorecognition

© 2013 WILEY-VCH Verlag GmbH & Co. KGaA, Weinheim

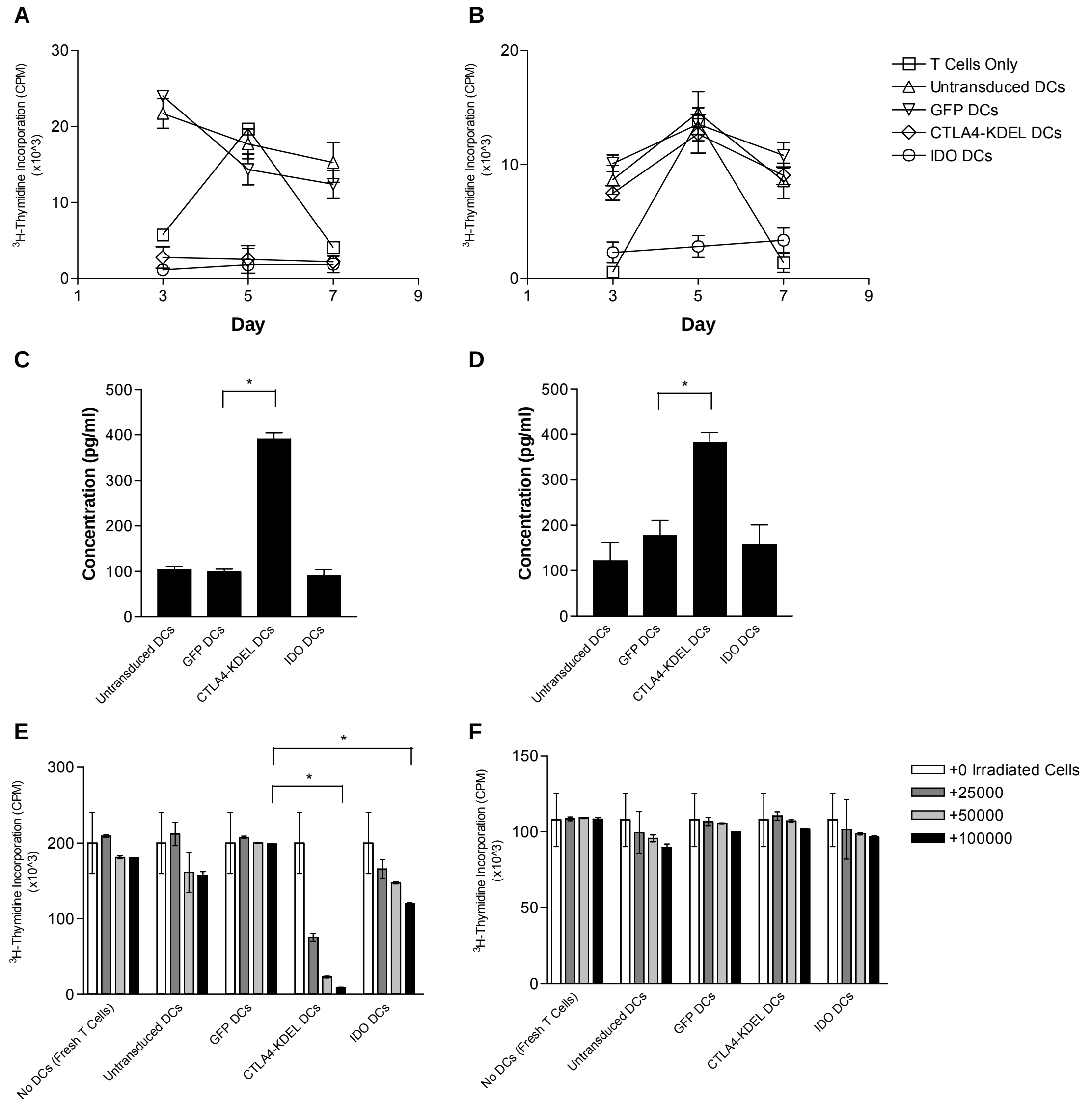

SUPPORTING FIGURE 1. Induction of donor alloantigen-specific T-cell anergy and regulation in vitro.

BALB/c DCs were transduced either with EIAV GFP (control), EIAV CTLA4-KDEL or EIAV IDO, followed by stimulation with LPS. The transduced (and untransduced) DCs were subsequently incubated in vitro with C3H/He-derived $CD4^+$ T cells. After 10 days, the T cells were harvested and rechallenged in vitro with **(A)** donor BALB/c DCs or **(B)** third-party C57BL/6 DCs, and $CD4^+$ T-cell proliferation assessed by thymidine incorporation on days 3, 5 and 7. **(C-D)** Culture supernatant from the donor DC rechallenge assay was harvested for detection of the immunoregulatory cytokines **(C)** TGF-β and **(D)** IL-10 by ELISA. **(E-F)** In addition, after 10 days of the primary DC - $CD4^+$ T-cell coculture, the T cells were harvested, irradiated and added (0-10^5 $CD4^+$ T cells) to a primary MLR between freshly-isolated C3H-derived $CD4^+$ T cells and **(E)** donor BALB/c DCs or **(F)** third-party C57BL/6 DCs. T-cell proliferation was assessed by thymidine incorporation after 5 days. All results are shown as the mean ± SD of triplicate wells and are representative of three independent experiments performed. * p<0.05, two-tailed *t*-test..

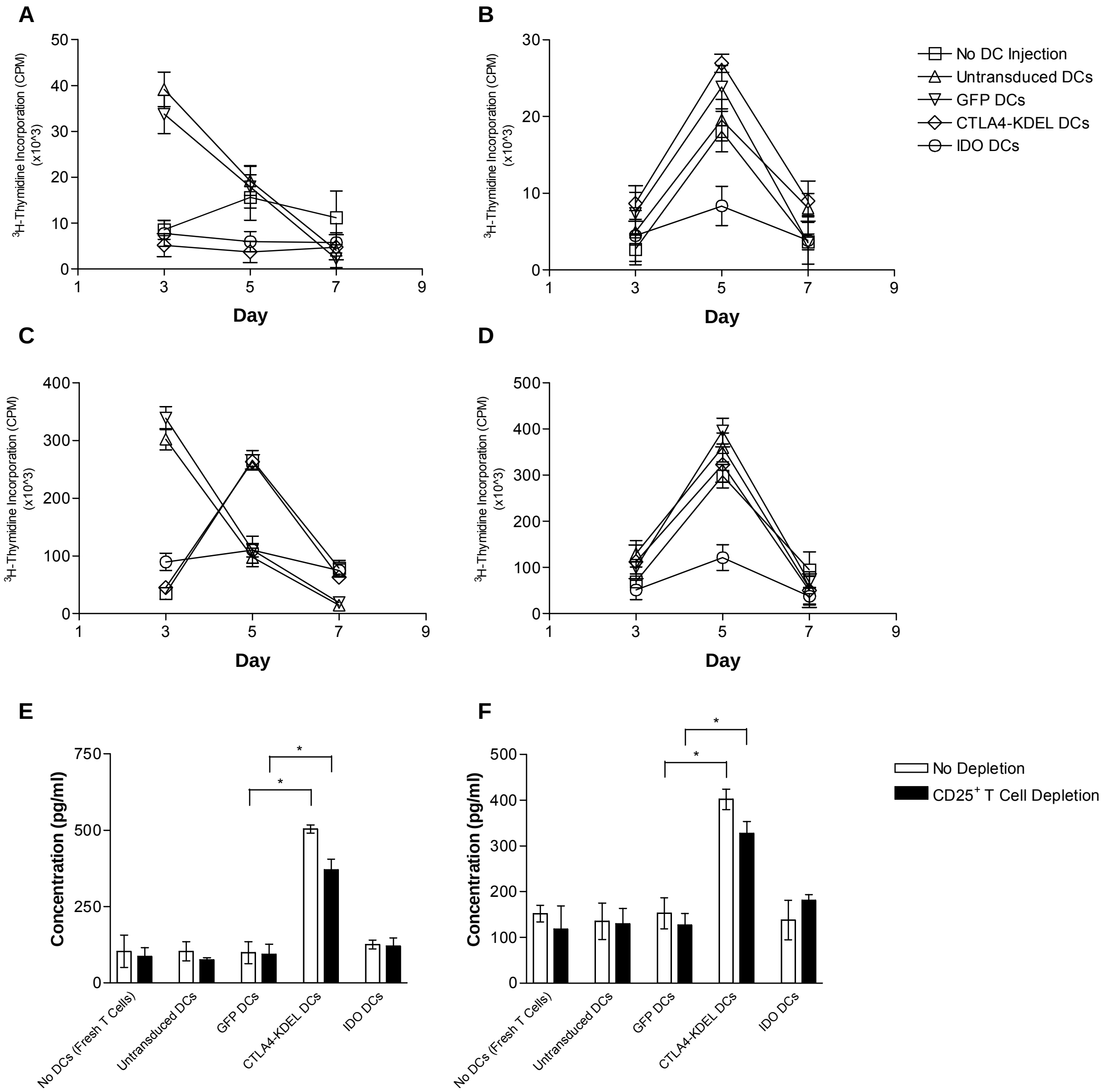

SUPPORTING FIGURE 2. Induction of donor alloantigen-specific T-cell anergy in vivo and production of immunoregulatory cytokines.

2.5×10^6 BALB/c DCs (either untransduced, or transduced with EIAV GFP (control), EIAV CTLA4-KDEL or EIAV IDO) were injected intravenously into C3H/He mice. **(A-D)** After 10 days, CD4$^+$ T cells purified from the spleens of injected mice were rechallenged in vitro with **(A)** donor BALB/c DCs or **(B)** third-party C57BL/6 DCs, and CD4$^+$ T-cell proliferation assessed by thymidine incorporation on days 3, 5 and 7. The purified CD4$^+$ T cells were also rechallenged with **(C)** donor BALB/c DCs or **(D)** third-party C57BL/6 DCs in the presence of 100 U/ml exogenous IL-2. **(E-F)** Culture supernatants from donor DC rechallenge assays (including those from CD4$^+$CD25$^+$ T-cell depleted-rechallenge assays) were harvested on day 7 for detection of the immunoregulatory cytokines **(E)** TGF-β and **(F)** IL-10 by ELISA. All results are shown as the mean ± SD of triplicate wells and are representative of three independent experiments performed. * p<0.05, two-tailed t-test.

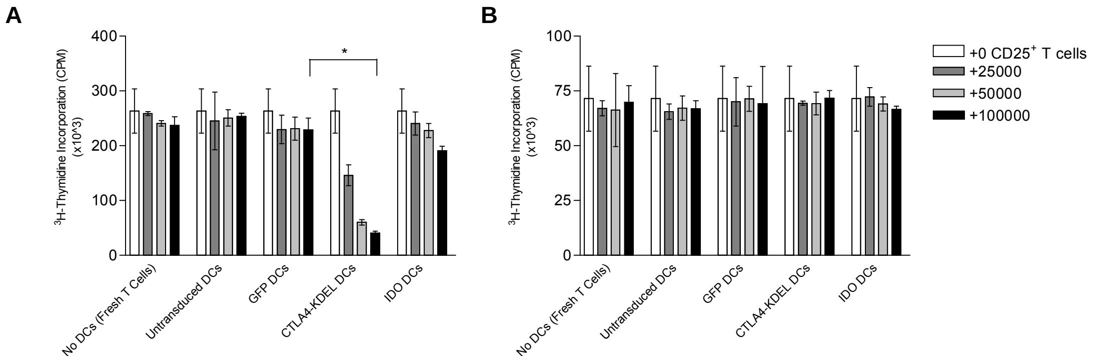

SUPPORTING FIGURE 3. Generation of donor alloantigen-specific Treg cells in vivo with capacity for linked suppression.

2.5x10^6 BALB/c DCs (either untransduced, or transduced with EIAV GFP (control), EIAV CTLA4-KDEL or EIAV IDO) were injected intravenously into C3H/He mice. **(A-B)** After 10 days, CD4$^+$CD25$^+$ T cells purified from the spleens of injected mice were irradiated and added in increasing numbers (0-10^5 CD4$^+$CD25$^+$ T cells) to a primary MLR between freshly-isolated C3H-derived CD4$^+$ T cells and **(A)** donor BALB/c DCs or **(B)** third-party C57BL/6 DCs. T-cell proliferation was assessed by thymidine incorporation after 5 days. All results are shown as the mean ± SD of triplicate wells and are representative of three independent experiments performed. * p<0.05, two-tailed t-test.

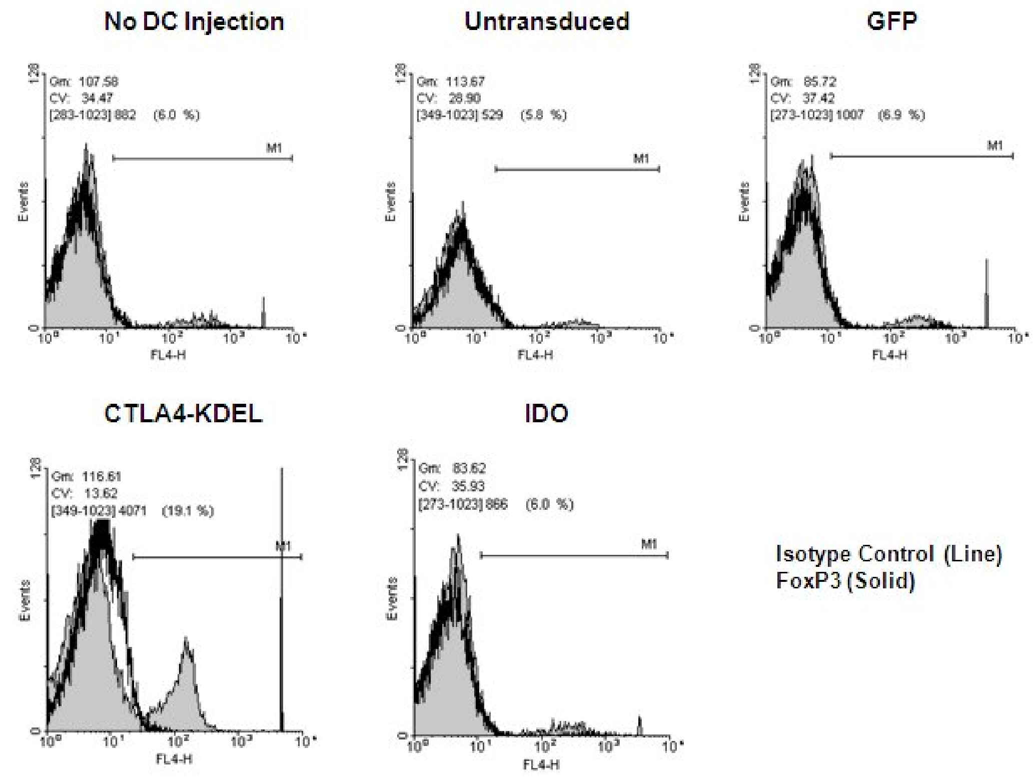

SUPPORTING FIGURE 4. Generation of FoxP3$^+$CD4$^+$CD25$^+$ T cells in vivo

2.5x10^6 BALB/c DCs (either untransduced, or transduced with EIAV-GFP (control), EIAV-CTLA4-KDEL or EIAV-IDO) were injected intravenously into C3H/He mice. After 10 days, CD4$^+$CD25$^+$ T cells purified from the spleens of injected mice were stained and analysed by flow cytometry for expression of FoxP3. The results shown in are representative of three independent experiments.
